# Supplementary material for: Healthcare Utilization and Costs in Sepsis Survivors in Germany–Secondary Analysis of a Prospective Cohort Study
Source: J Clin Med. 2022 Feb 21;11(4):1142. doi: 10.3390/jcm11041142 (PMC8879304; doi:10.3390/jcm11041142)
Supplement: Supplementary file 1 [file jcm-11-01142-s001.zip › jcm-1556903-proofed-suppl.pdf]

Table S1. Description of costing dataset.

Inpatient utilization		Costs per unit [€]
Hospitalization	Costs/ days of inpatient treatment (including ICUs and psychiatric hospitals) assessed by standardized cost unit rates according to Bock et al. [1]	593.04
Rehabilitation	Costs/ days of inpatient rehabilitative care (excluding outpatient rehabilitative care) assessed by standardized cost unit rates according to Bock et al. [1]	121.85
**Outpatient utilization**		**Costs per unit [€]**
PCP visits	Mean costs per PCP contact, being defined as total of medical fees/ number of contacts [3]	20.06
Specialty visits	Mean costs per contact, being defined as total of medical fees/ number of contacts [3]	
	Internal specialists	65.44
	Surgeons	43.39
	Urologists	24.70
	Neurologists/Psychiatrists	44.72
	ENT specialists	26.40
	Ophthalmologists	34.78
	Dermatologists	18.89
	Orthopedists	25.42
	Psychotherapists	78.08
Medication	Costs were estimated using the "Lauer-Taxe" database [2]. When not specified, a package size of N3 was assumed, equating a quantity for a treatment of 100 days.	
Diagnostic Testing	Costs were estimated using the reimbursement scheme provided by the National Association of Statutory Health Insurance Physicians [3]. Costs for body-imaging were taken from the EBM of Radiology, for non-imaging diagnostics the EBM schemes of the particular specialties (e.g. Internal Medicine, Neurology, Nuclear Medicine) were used.	
Allied Health Visits	Standard value of contact according to the "GKV-Heilmittelinformationssystem"[4]	
	Occupational therapy	37.51
	Physiotherapy	16.42
	Speech therapy	38.59
	Podologist therapy	27.51
Durable Medical Equipment (DME)	Valuation rates according to the Remedy and Aid report of the Barmer GEK health insurance [5]	
	Walking frames	52.74
	Utilities for elastic compression	140.66
	Utilities for incontinence treatment	346.43
	Vehicles	720.49
	Utilities for bathing/showering	153.37

Table S2: Prescription of analgesics and antidepressants, comparisons between groups with both cross sectional data available at three months before sepsis and six, 12 and 24 months post ICU respectively.

	**3 months before sepsis**	**6 months post ICU**
**Analgesics**	*n* = 194 (missing: 30)	*n* = 194 (missing: 12)
≥one prescription, No. (%)	35 (21.3%)	53 (29.1%)
no prescription, No. (%)	129 (78.7%)	129 (70.9%)
**Antidepressants**	*n* = 194 (missing: 30)	*n* = 194 (missing: 12)
≥one prescription, No. (%)	11 (6.7%)	35 (19.2%)
no prescription, No. (%)	153 (93.3%)	147 (80.8%)

	**3 months before sepsis**	**12 months post ICU**
**Analgesics**	*n* = 177 (missing: 31)	*n* = 177 (missing: 12)
≥one prescription, No. (%)	32 (23.7%)	53 (30%)
no prescription, No. (%)	114 (64.4%)	112 (63.3%)
**Antidepressants**	*n* = 177 (missing: 31)	*n* = 177 (missing: 12)
≥one prescription, No. (%)	11 (6.2%)	24 (13.6%)
no prescription, No. (%)	135 (76.3%)	141 (79.7%)

	**3 months before sepsis**	**24 months post ICU**
**Analgesics**	*n* = 146 (missing: 25)	*n* = 146 (missing: 28)
≥one prescription, No. (%)	27 (18.5%)	43 (29.5%)
no prescription, No. (%)	94 (64.4%)	75 (51.4%)
**Antidepressants**	*n* = 146 (missing: 25)	*n* = 146 (missing: 28)
≥one prescription, No. (%)	8 (5.5%)	20 (13.7%)
no prescription, No. (%)	113 (77.4%)	98 (67.1%)

Table S3. Change in single cost categories between short - and long-term post-ICU resource utilization.

	**0–6 Months**				**13–24 Months**				**Difference (Mean)**	**Difference (Median)**	**Difference (IQR)**	**Percentiles (25; 75)**	***p*-Value**
	Mean	***SD***	***Median***	***IQR***	***Mean***	***SD***	***Median***	***IQR***					
**Rehospitalisation: Costs per patient (€)**	13786.6	23075.86	2965.2	17791.2	5114.41	10580.86	0.00	5930.40	8672.19	0.00	10229.94	(−1186.08; 9043.86)	0.001
Rehospitalisation: average length of stay (d)	23.25	38.91	5.00	30.0	8.62	17.84	0.00	10.00	14.63	0.00	17.25	(−2.00; 15.25)	0.001
**Rehabilitative Care: Costs per patient (€)**	1591.37	2195.46	0.00	2680.7	311.79	1053.51	0.00	0.00	1279.58	0.00	2558.85	(0.00; 2558.85)	0.000
**Medication costs per patient (€)**	143.66	383.19	0.00	65.25	48.65	235.35	0.00	0.00	95.01	0.00	46.8	(0.00; 46.80)	0.012
Antidepressants: Costs per patient (€)	17.76	56.07	0.00	0.00	2.12	14.15	0.00	0.00	15.62	0.00	0.00	(0.00; 0.00)	0.084
Analgesics: Costs per patient (€)	125.91	376.94	0.00	45.00	46.53	234.44	0.00	0.00	79.38	0.00	22.14	(0.00; 22.14)	0.010
**(All) allied health visits: Costs per patient (€)**	177.90	218.26	164.2	164.2	139.88	209.30	0.00	164.20	38.02	0.00	164.2	(0.00; 164.20)	0.109
**Medical aids: Costs per patient (€)**	234.12	503.67	0.00	193.4	230.36	538.39	0.00	140.66	3.76	0.00	0.0	(0.00; 0.00)	0.655

References

1. Bock, J.O.; Brettschneider, C.; Seidl, H.; Bowles, D.; Holle, R.; Greiner, W.; König, H.H. Calculation of standardised unit costs from a societal perspective for health economic evaluation. *Gesundheitswesen* **2015**, *77*, 53–61.
2. All medication costs from LAUER-Taxe online-database (standard reference book for pharmaceuticals) LAUER-Taxe online-database, Lauer-Fischer GmbH; WEBAPO® InfoSystem, 2016. Available from: https://www.cgm.com/deu_de/produkte/apotheke/lauer-taxe.html (accessed on 9 February 2022).
3. Costs for diagnostics the EBM (Einheitlicher Bewertungsmaßstab), reimbursement scheme provided by the „Kassenaerztliche Bundesvereinigung“ (National Association of Statutory Health Insurance Physicians), Berlin, Germany Bundesvereinigung KK. Arztgruppen-EBM Radiologie. 2016. Available from: https://www.kbv.de/html/85.php (accessed on 9 February 2022).
4. GKV Spitzenverband: Heilmittel-Schnellinformation nach § 84 Abs. 5, i. V. m. Abs. 8 SGB V – Bundesbericht 2011: https://www.gkv-heilmittel.de/media/dokumente/his_statistiken/2011_04/HIS-Bericht-Bund_201104.pdf (accessed on 9 February 2022).
5. Kemper, C.; Sauer, K.; Glaeske, G. *BARMER GEK Heil- und Hilfsmittelreport 2012;* BARMER GEK: Schwäbisch Gmünd, Germany, September, 2012.
