# Supplementary figures and images for: Healthcare Utilization and Costs in Sepsis Survivors in Germany–Secondary Analysis of a Prospective Cohort Study
Source: J Clin Med. 2022 Feb 21;11(4):1142. doi: 10.3390/jcm11041142 (PMC8879304; doi:10.3390/jcm11041142)

**Fig. S1A**

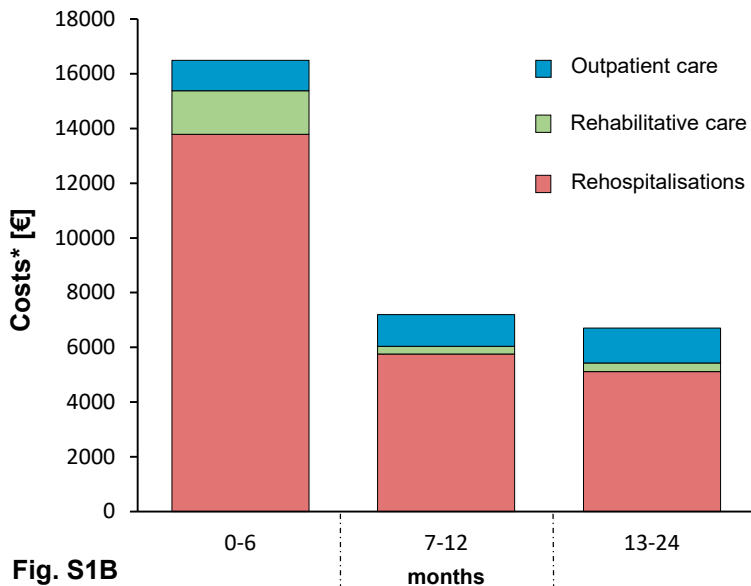

**Fig. S1B**

Outpatient cost details

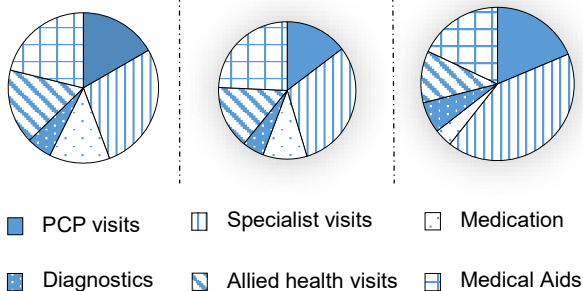

Supplement: Supplementary file 1 [file jcm-11-01142-s001.zip › 202202_Ressource use_post_sepsis_Fig_S1.pdf]
